# Providing Unique Support for Health Study Among Young Black and Latinx Men Who Have Sex With Men and Young Black and Latinx Transgender Women Living in 3 Urban Cities in the United States: Protocol for a Coach-Based Mobile-Enhanced Randomized Control Trial

**DOI:** 10.2196/17269

**Published:** 2020-09-16

**Authors:** Renata Arrington-Sanders, Kimberly Hailey-Fair, Andrea Wirtz, Travis Cos, Noya Galai, Durryle Brooks, Marne Castillo, Nadia Dowshen, Constance Trexler, Lawrence J D’Angelo, Jennafer Kwait, Chris Beyrer, Anthony Morgan, David Celentano, PUSH Study

**Affiliations:** 1 Division of Adolescent and Young Adult Medicine Johns Hopkins School of Medicine Baltimore, MD United States; 2 Department of Epidemiology Johns Hopkins Bloomberg School of Public Health Baltimore, MD United States; 3 Public Health Management Corporation Research & Evaluation Group Philadelphia, PA United States; 4 Department of Statistics University of Haifa Mt Carmel Israel; 5 The Adolescent Initiative Children's Hospital of Philadelphia Philadelphia, MD United States; 6 Craig Dalsimer Division of Adolescent Medicine Children's Hospital of Philadelphia Philadelphia, MD United States; 7 Adolescent Clinical Research Burgess Clinic Children's National Medical Center Washington, DC United States

**Keywords:** African-American, Latinx, men and transgender, HIV, youth, mobile phone

## Abstract

**Background:**

The US National HIV/AIDS Strategy 2020 calls for increasing access to care, improving outcomes of people living with HIV, and targeting biomedical prevention efforts, including access to pre-exposure prophylaxis (PrEP) in communities where HIV is most heavily concentrated. The cities of Baltimore, Maryland (MD); Washington, DC; and Philadelphia, Pennsylvania (PA) are disproportionately burdened by high rates of new cases of HIV infection, with high prevalence among young Black and Latinx men who have sex with men (YBLMSM) and young Black and Latinx transgender women (YBLTW) aged 15-24 years.

**Objective:**

This study aims (1) to identify and recruit YBLMSM and YBLTW who are at risk or living with HIV in Baltimore, MD; Philadelphia, PA; and Washington, DC, using respondent-driven sampling (RDS) with targeted seed selection, and (2) to assess the efficacy of a coach-based mobile-enhanced intervention (MEI) compared with standard of care (SOC) to increase successful engagement and retention into HIV, PrEP, and substance use treatment care across the HIV care and prevention continua in 3 Mid-Atlantic cities. This paper describes the protocol and progress as of October 20, 2019.

**Methods:**

This study uses a multiphase mixed methods design. The first phase is a formative, qualitative research with focus group discussions and key informant interviews. The second phase consists of evaluating the ability of RDS with targeted seed selection. The third phase includes 2 embedded randomized controlled trials (RCTs), where participants complete a baseline sociobehavioral survey, rapid HIV testing, and eligible youth enroll in parallel status-dependent RCTs that randomize the participant to 1 of 2 study arms: MEI with coach or SOC. Participants are asked to complete a web-based survey and provide biologic specimens—HIV-1 RNA (viral load) or HIV-1 antibody test and urine drug screen—at baseline and at 3, 6, and 12 months, and an exit interview at 18 months.

**Results:**

A formative qualitative research was conducted in February 2017 and May 2018, and this led to further refinement of recruitment and study methods. Aim 1 recruitment began in September 2017 with subsequent enrollment into the RCTs. Recruitment is ongoing with 520 participants screened and 402 (77.3%) enrolled in aim 1 by October 2020. Of these, 159 are enrolled in the 2 randomized trials: 36 (22.6%) HIV-positive not virally suppressed (aim 2) and 123 (77.4%) high-risk HIV-negative (aim 3).

**Conclusions:**

This study has the potential to significantly impact the medical and substance use services provided to YBLMSM and YBLTW in the United States by providing rigorous scientific evidence outlining approaches and strategies that improve the uptake and engagement of YBLMSM and YBLTW in the HIV treatment and prevention continuum.

**Trial Registration:**

ClinicalTrials.gov NCT03194477; https://clinicaltrials.gov/ct2/show/NCT03194477

**International Registered Report Identifier (IRRID):**

DERR1-10.2196/17269

## Introduction

### Background

The US National HIV/AIDS Strategy 2020 [1] calls for (1) increasing access to care and improving outcomes of people living with HIV and (2) targeting biomedical prevention efforts, including access to pre-exposure prophylaxis (PrEP) in communities where HIV is most heavily concentrated. The cities of Baltimore, Maryland; Washington, DC; and Philadelphia, Pennsylvania, are disproportionately burdened by high rates of new cases of HIV infection, with highly elevated rates among young Black and Latinx men who have sex with men (YBLMSM) and young Black and Latinx transgender women (YBLTW) aged 15-24 years [2-5]. This underscores the need for increased identification, linkage, and initiation in HIV treatment and preventive care for these populations.

Strategies to change the trajectory of the epidemic for those most affected now focus on status-based cascade approaches that seek to systematically connect individuals at different stages within the HIV continuum to different stages of prevention and treatment to reduce their likelihood of acquiring HIV, and for those with HIV, achieving viral suppression [6-8]. The cascade approach seeks to identify one’s HIV infection status and immediately engage the individual in HIV prevention, typically using PrEP, or in HIV care. Although earlier engagement in the HIV care cascade can improve the overall outcomes, YBLMSM and YBLTW disproportionately fall out of the treatment cascade at an early stage [9]. Both YBLMSM and YBLTW are less likely than White young men who have sex with men (MSM) or their White cisgender peers to receive, adhere, and obtain HIV viral suppression with antiretroviral therapy (ART) [10,11]. Moreover, YBLMSM and YBLTW at risk for HIV have had disproportionately lower rates of PrEP uptake and adherence [3,12,13].

High rates of substance use [14] in YBLMSM and YBLTW have been identified as key factors that influence treatment and prevention engagement [15-17]. YBLMSM and YBLTW with substance use disorders (SUDs) and *risky* substance use may be less likely to perceive a need for treatment and more likely to experience barriers to engaging in care [18,19]. Findings in other research indicate that behavioral interventions should assess substance use to inform clinical guidance in education and medical consultation for young MSM [20]. Related anxiety and other mental health diagnoses, which are often associated with substance use, may also be relevant to informing an intervention to reduce risk behavior [21]. As such, substance use interventions may increase the effectiveness of HIV treatment and prevention programs [22].

Communities of color and low-income communities often experience limited access to HIV care, substance treatment, and lesbian, gay, bisexual, transgender, queer (LGBTQ) services [19,23-26]. Multiple barriers have been identified, including barriers related to race, sexual identity, gender identity, and intersectional discrimination or stigma experienced from existing within multiple marginalized identities [27-30]. Concerns about confidentiality and mistrust have also been identified as barriers for youth [31]. Youth-based interventions that are accessible and simultaneously address HIV care, prevention, and substance use treatment behaviors are needed, yet few exist [14].

One strategy that has shown promise in identifying hard-to-reach MSM and transgender women (TW) at risk of HIV and providing them with the opportunity to receive PrEP, early ART, and substance use is a peer-driven recruitment [32]. Peer-driven recruitment has been identified as an effective strategy to reach MSM, including young Black MSM, and TW at high risk of HIV in the United States and Thailand [33,34]. In both studies, peer recruitment resulted in earlier access to HIV and prevention services, including PrEP and early ART [32,34]. Respondent-driven sampling (RDS) is a peer recruitment strategy that has been used to identify and engage hard-to-reach HIV-infected but untreated Black MSM in HIV care [32,35,36]. Typically, RDS involves a chain-referral procedure in which well-networked individuals (seeds) are asked to invite a number of peers from a specified population to be screened for a study. The *seeds* are often given a certain number of enrollment slips or coupons to provide to their peers. YBLMSM and YBLTW social networks are likely to overlap. However, limited studies have sought to use RDS to recruit these overlapping populations. Tailored strategies for youth, such as incorporating RDS *seeds* from social networks, may improve the identification and recruitment of HIV-infected and high-risk HIV-uninfected YBLMSM and YBLTW and identify individuals with comorbid factors, including substance use, that may impact engagement and retention in care and prevention [37].

With 94% of Black teens and 99% of lesbian, gay, bisexual, and transgender young adults owning or having access to a smartphone [38], mobile-based interventions have the potential to engage youth in HIV prevention and the treatment cascade [39]. The benefits of mobile apps to engage gay and bisexual men have been documented by other researchers [40-42]. However, few have tried to simultaneously address both the HIV prevention and treatment cascade and substance use behaviors that may coexist among YBLMSM and YBLTW. Further, engagement in prevention and treatment has been a challenge even for prior studies that have effectively demonstrated short-term changes in condom use behavior [43]. Peer support (or coaching) with a mobile app has the potential to improve engagement through interactive motivational feedback around HIV and substance use risk. Such approaches have the capacity to screen adolescents for HIV risk and provide brief substance use interventions that are a flexible, low-intensity strategy that empowers youth and connects them to earlier access to HIV treatment, prevention services, and reduction of specific risks identified. This may be prudent given the prior studies that have demonstrated that community-based mentoring and peer support effectively build resilience and address barriers to care, including stigma and homophobia in YBLMSM and YBLTW [44-46]. To date, however, few studies have examined such approaches among YBLMSM and YBLTW living in the United States.

### Objectives

The goal of this paper is to describe a study protocol to (1) identify and recruit YBLMSM and YBLTW who are at risk or living with HIV in Baltimore, MD; Philadelphia, PA; and Washington, DC, and (2) to assess the efficacy of a coach-based mobile-enhanced intervention (MEI) compared with standard of care (SOC) to increase successful engagement and retention in HIV care, prevention, and substance use treatment for YBLMSM and YBLTW living in the 3 Mid-Atlantic cities. Ultimately, we are interested in using the HIV care and prevention continuum [2,47] to evaluate strategies to identify and engage YBLMSM and YBLTW who are at risk or living with HIV, while simultaneously addressing substance use comorbidity [48]. The manuscript focuses on the development and formative work of this study and a summary of the protocol.

The research protocol described here is a collaboration between the Johns Hopkins Schools of Medicine (Harriet Lane Clinic) and Bloomberg School of Public Health, the Public Health Management Corporation, and 3 collaborating sites: Children’s Hospital of Philadelphia (CHOP), Children’s National Hospital, and Whitman-Walker Health.

## Methods

### Study Design

This study uses a multiphase mixed methods design [49]. The first phase is an exploratory, incorporating formative, qualitative research with key informant interviews (KIIs) and focus group discussions (FGDs). These KIIs and FGDs are intended to inform further development and refinement of the methods utilized in the study, including questionnaire content, components of the intervention, and topics explored in the embedded qualitative research [50].

**Figure 1:**
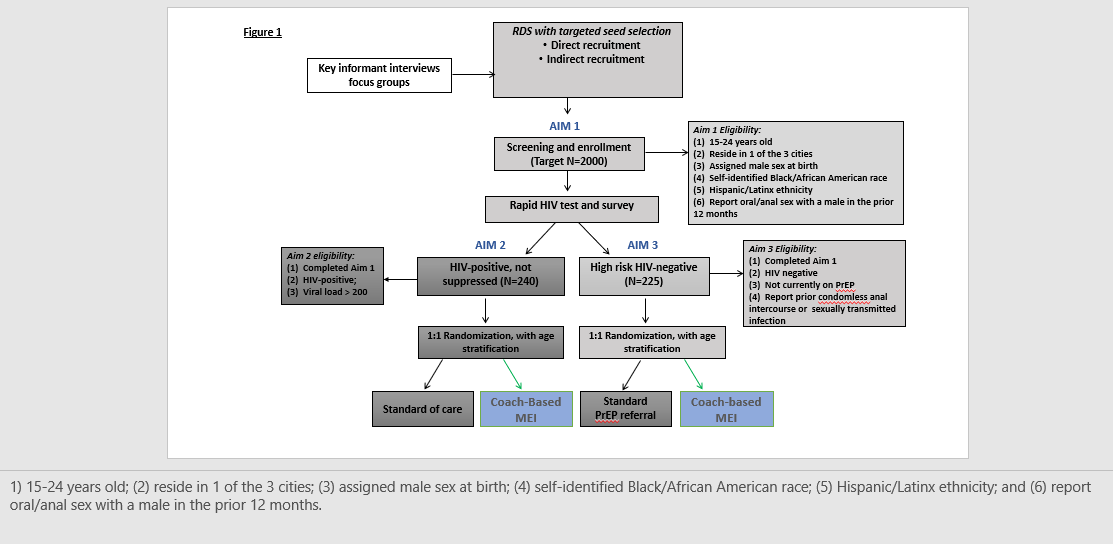
Study flow diagram. MEI: mobile-enhanced intervention; PrEP: pre-exposure prophylaxis; RDS: respondent-driven sampling.

The protocol covers the 3 aims of the study. The first aim is to identify and recruit YBLMSM and YBLTW living in the 3 Mid-Atlantic cities into the prevention and treatment continua. The second aim is to use an embedded randomized controlled trial (RCT) to examine the efficacy of a coach-based MEI to achieve sustained retention and engagement in HIV care among youth living with HIV who are not virally suppressed. The third aim is to use an embedded RCT to examine the efficacy of a coach-based MEI to achieve uptake of and adherence to PrEP and other preventive behaviors (eg, *protected* sex acts) among youth at elevated risk for HIV infection. Both aims 2 and 3 examine identification, referral, and engagement in substance treatment services. The outline of the study design is shown in Figure 1.

### Study Setting

The study is being conducted in the following US cities: Baltimore, MD (Johns Hopkins University); Philadelphia, PA (CHOP); and Washington, DC (Children’s National Hospital and Whitman-Walker Health). Each city experiences high rates of HIV among YBLMSM and YBLTW (aged 15-24 years) [3].

### Formative Research Focus Groups and KIIs

Focus groups and KIIs were used in the formative phase for the purpose of adapting the study tools, identifying key components of the intervention, and developing the intervention structure. The formative phase was conducted between February 2017 and May 2018. First, 3 focus groups were conducted in Baltimore, MD, to modify and adapt an app for the MEI to meet the needs of YBLMSM and YBLTW. Following focus groups, key informants (KIs) were interview-identified across all 3 cities to inform the refinement of the interventions proposed by the focus groups and to identify topics to be explored in the qualitative interviews.

### Formative Research Study Sample

Participants in the focus groups were eligible to participate if they currently resided or worked in Baltimore, MD, were aged 15 to 24 years, and either self-identified as YBLMSM or YBLTW. Participants of focus groups were recruited through flyer advertisements or provider referrals. KIs and focus group participants were reimbursed US $35 for participating. KIs included young adults with either a social or mentoring role in the community or someone who worked as a case manager, community outreach specialist, or volunteer. KIs were eligible to participate if they currently resided or worked in Baltimore, MD; Philadelphia, PA; or Washington, DC, and were identified by LGBTQ-serving organizations as persons who were in or around the target age range (18-24 years) and served as YBLMSM and YBLTW or a part of the community. Organizations who referred to KIs were asked to confirm that the KIs fit the eligibility criteria of the study.

### Data Collection for Formative Research

**Figure 2:**
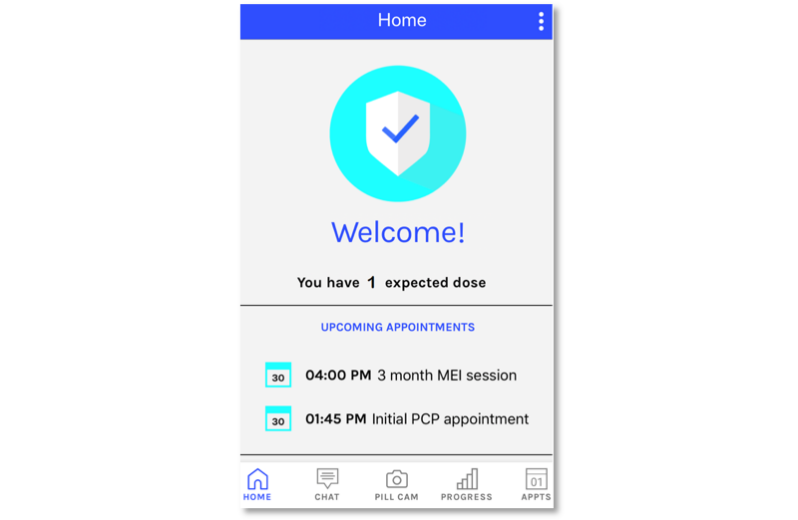
Wireframe of mobile app. MEI: mobile-enhanced intervention; PCP: Primary Care Provider.

The 3 focus groups lasted for 45 to 60 min and occurred in Baltimore, MD, only, with an average of 3 participants per group. Facilitation of the focus groups was done in collaboration with emocha Health Inc, a Baltimore-based company that assisted with the development of the mobile app. The goal of the focus groups was to identify key areas of focus for the intervention, mobile app components, and to elicit feedback on wireframes (Figure 2). Wireframes were based on areas developed by emocha Health, Inc for other adherence studies at a seventh-grade literacy level or lower [51]. Additional feedback was provided by an established sexual minority youth advisory board at the Philadelphia site.

KIs elicited feedback about the intervention from youth or service providers from all 3 cities. The one-time, face-to-face, video or telephone interview lasted approximately 45 min. Semistructured interview guides were created to facilitate discussion around the topics of community and societal barriers to accessing HIV treatment and prevention among YBLMSM and YBLTW and strategies (eg, navigator, coach, and mobile app) that could be used to address barriers. Participants were then asked to provide feedback on specific components of the mobile app developed by emocha Health, Inc around potential use and functionality. Example questions used in the focus groups and KIs included, “Rank the following features that would be helpful to you (or a YBLMSM or YBLTW aged 15 to 24 years) in improving your (their) health and following up with your (their) medical care: (1) goal planning for medications, (2) adherence reminders, (3) adherence support (rewards or points for positive adherence, coach support), (4) pill cam, (5) progress calendar; (6) personalized health information or text messages, and (7) general information (information about testing, condoms, resources, housing, and insurance). Why did you select (insert top choice) as your top choice?”

### Data Analysis

Focus groups and interviews were audio-recorded and transcribed verbatim by an independent transcription company. Initial codes were identified from the interview and focus group guides developed by our research team. The codes were then refined and elaborated during the process of analysis using the constant comparison method [52].

Using a systematic approach to further validate and connect categories continued until the point of data saturation [53]. Grouped and categorized codes were then examined for emergent themes [49]; approximately 10% (n=3) of transcripts were double-coded and then reviewed for intercoder reliability or consistency to ensure high coder agreement (Kappa>0.80). Transcripts were reviewed using NVivo 11 software and included analyses based on the presence of identified codes and emergent concepts.

A total of 10 youth participated in the focus groups, and 18 KIs participated in formative interviews. Focus group participants suggested that the app include the following components: (1) goal planning for ART adherence and substance use treatment, (2) time reminders, (3) adherence reminders, and (4) adherence support (rewards or points for achieving optimal adherence). Findings from key interviews have been described elsewhere [54]; however, KIs recommended that the study allow for a longer follow-up time (greater than 4 weeks) for coupons to ensure engagement around recruitment, and suggested that the app has rewards for achievement. KIs suggested that the intervention focused on values (health priorities and personal goals), medication initiation and adherence, medical appointments, alcohol or drug use, and sexual health. The KIs suggested that the intervention should be flexible with bidirectional support with a coach, using multiple methods (eg, app, text message, and telephone calls), and the coach must be a peer or close to the population to enhance engagement with the intervention.

### Clinical Trial Methods

#### Aim 1

##### Sample and Setting

The first aim of this protocol is to identify and recruit YBLMSM and YBLTW who are at risk or living with HIV in Baltimore, MD; Philadelphia, PA; and Washington, DC, using RDS with targeted seed identification. This protocol focuses on participants who are (1) aged 15 to 24 years, (2) reside in the 3 study cities, (3) assigned male sex at birth, (4) self-identified Black/African American race or Hispanic/Latinx ethnicity, and (5) report oral or anal sex with a male in the prior 12 months. We included youth assigned male sex at birth, including both transgender and gender-expansive youth as well as nonbinary youth, given the gender diversity, dynamic, and fluidity of gender identity during adolescence. In addition, adolescents may be at varying places along the gender transition spectrum and may or may not identify as male or transgender [55].

##### Recruitment

**Figure 3:**
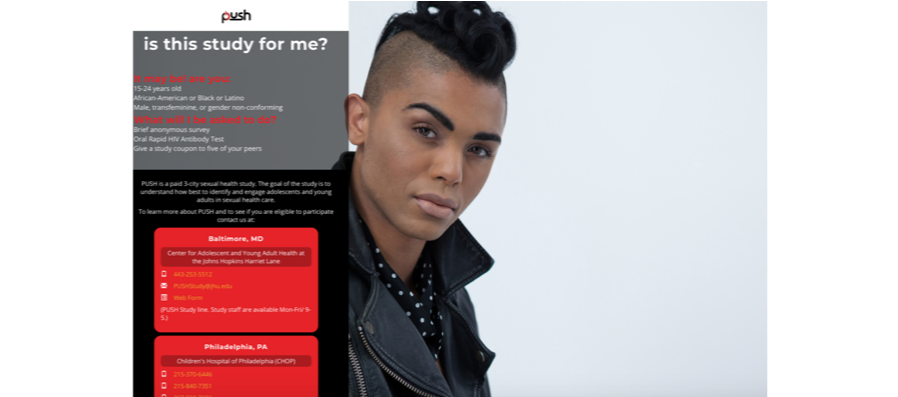
Web-based Flyer.

**Figure 4:**
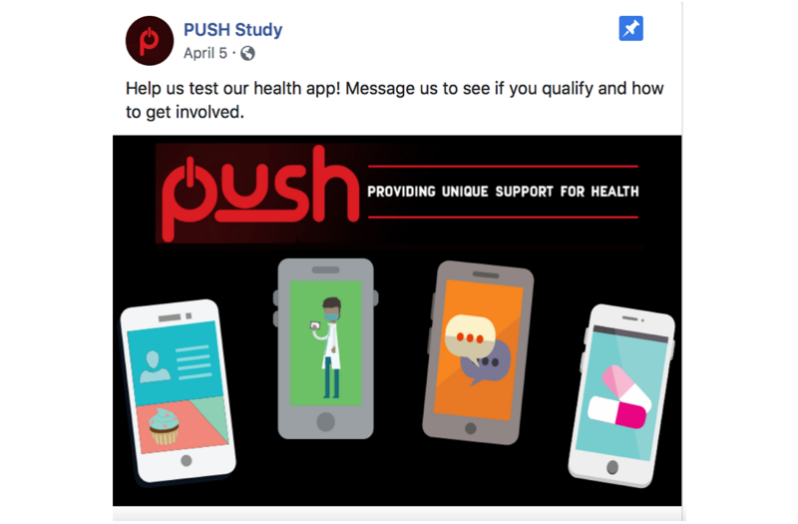
Example of Facebook/Instagram.

Participants are recruited using RDS with targeted seed selection. As part of aim 1, we aimed to compare direct and indirect targeted seed selection methods for reaching at-risk youth (behaviorally at risk for HIV acquisition or living with HIV and virally unsuppressed). Direct seed selection consists of informational flyers directly given to youth from study staff at a clinical site or at a community-based organization or in-person event (eg, house ball, pride, or community informational session). Indirect seed selection consists of informational flyers posted at clinical sites that serve LGBTQ youth or at the offices of community-based organizations. Indirect seed selection also includes a web-based approach that consists of electronic advertisements placed on webpages and social and sexual networking sites frequented by YBLMSM (eg, Jack’d, Black Gay Chat, and Grindr; Figures 3 and 4). A link on the web-based advertisements directs potential participants to the study landing page where they can leave their information for staff to contact them about participating [56]. The staff maintain the page and rotate images frequently to maintain engagement. Staff return all web messages within 48 hours.

RDS is a chain-referral sampling method that is typically used to approximate a probability sample of populations when sampling frames are unavailable [57,58]. For the purpose of this protocol, RDS is used to recruit behaviorally at-risk youth to status-dependent RCTs. Each seed was asked to participate in the study and to invite up to 5 peers from the target population to be screened for study eligibility. Unlike other HIV research, our goal is not to approximate a probability sample to produce population-based estimates but to investigate associations with risk behaviors and to identify participants who could be enrolled in the RCTs.

As recommended during formative research, this study uses electronic coupons (e-coupons) to facilitate the distribution of study coupons, given the challenges youth may face in physically meeting with peers to distribute paper coupons (as had been done previously). At the initial visit, a staff member meets with a participant, brainstorms about who to distribute coupons to peers, and provides instructions for the distribution of e-coupons using cell phones. The staff member provides a unique participant web link to the participant, which they then use to send their e-coupon to peers, including friends and social contacts. The link takes the participant to a page where they can see and manage the sharing of their e-coupons with peers. During the visit, the participant is asked to send their e-coupons to their peers (electronic respondent-driven sampling [eRDS] recruit) and to verify receipt. The eRDS recruit (a person receiving the e-coupon) receives a text message informing them that they have been invited to participate in a research study. The text message includes a unique numeric code, study telephone number, study site operating hours, and an expiration date (approximately 2 weeks after issuance). The recruit then contacts the study team to set up an appointment at the study site, at which point they are asked to provide their e-coupon to staff to initiate eligibility screening. The unique numeric code of the e-coupons permits the identification of linkages between *seeds* or recruiters and recruits in the data. E-coupons omit any sensitive information about participating in an HIV-related study, though *seeds* or recruiters and peers can discuss this verbally. Participants who are unable to distribute e-coupons or who choose not to use e-coupons are provided paper coupons for distribution.

##### Study Activities

Participants, including both *seeds* and recruits, are provided the opportunity to complete a survey and a rapid HIV testing (OraQuick Advance or INSTI). The survey includes measures of key demographics, social network characteristics, HIV testing and diagnosis history, substance use, and history of PrEP use (Textbox 1). Skip patterns are used to reduce the number and complexity of the questions. All data measures are captured in the same data system that is designed for RDS data collection and tracking. Data are tracked with a unique study ID only and are not associated with participant contact information.

Participants are asked to return in 2 weeks for coupon payment, to complete a second survey that asks about their social network and in what context coupons are provided, and to participate in a booster RDS training where they spend additional time with staff reviewing techniques and strategies to promote the distribution of coupons [59]. Participants are reimbursed US $5-15 per peer recruited peer (dependent on site).

**Study domains and measures.**

**Demographic measures**

- Age, gender identity, marital status, education, annual income, race and ethnicity, health insurance status, housing, and employment status

**Respondent-driven sampling**

- Network size and characteristics, utilized for respondent-driven sampling weighting

**Internet or mobile phone use**

- Type of phone, data plan, and frequency of use; loss of plan; and other family members who have access to the phone

**Venue use**

- Information on physical venues where participants frequent will be collected; venues will be included to assess overlap between physical and web-based seed networks

**HIV testing and care history**

- Frequency of HIV testing in the past year, date of most recent HIV test, and receipt of HIV test results

**HIV continuum**

- Self-reporting HIV infection; current engagement in care, including antiretroviral treatment; adherence; and self-reported viral load status

**Knowledge of pre-exposure prophylaxis (PrEP) and postexposure prophylaxis (PEP)**

- Knowledge, perception of, and history of PrEP and PEP use

**Sexual matrix module and sexual risk behavior**

- Sexual attraction, identity, and behavior; 7-item question HIV Incidence Risk Index for men who have sex with men (MSM) males only [60,61]; information on number of partners and frequency and types of sexual acts; network of the sexual contacts; location of where sexual experiences occurred; and perceived sexual risk for sexually transmitted infections, including risk for HIV

**Health care use**

- Health care and services utilization and engagement and trust between the clinician and patient

**Stigma and mental health**

- Self-stigma (internalized homo-negativity) and HIV stigma (with consideration of impact on HIV testing and adherence) adapted from Herek et al [62,63]; mental health will include lifetime and recent history of depressive symptoms and anxiety, using the Patient Health Questionnaire-4 [64], post-traumatic stress disorder (Child Post-Traumatic Stress Disorder Symptom Scale) [65], and experiences of discrimination [66]

**Social environment**

- Outness Inventory [67]; intimate partner violence, victimization, and perpetration (Safe Dates instrument) [68]; sexual guilt (Revised Mosher Sex-Guilt Scale) [69]; lesbian, gay, bisexual, transgender, and questioning (LGBTQ) victimization [70]; and social context (perceived parental support and religious environment) [71]

**Substance use**

- Car, relax, alone, forget, friends, and trouble (CRAFFT) six-item substance use screen and a modified Time Line Followback assessing days of use of various substances [72,73]

**Engagement in the lesbian, gay, bisexual, transgender, queer (LGBTQ) community**

- Measures of strength of engagement and connection in the LGBTQ community

#### Coach-Based MEI for Youth Living With HIV

The second aim of the protocol is an RCT to examine the efficacy of a mobile-enhanced coach-based intervention to achieve sustained retention and engagement in HIV care among youth living with HIV who are not virally suppressed.

#### Aim 2

##### Study Sample

YBLMSM or YBLTW, recruited into aim 1, who are identified as HIV-positive (previously diagnosed with HIV—self-report of HIV-1 and confirmed viral load, or newly diagnosed—no prior history of HIV and rapid HIV test positive) are invited to participate in the RCT of aim 2 of the study. Additional eligibility includes not virally suppressed; no plan to relocate in the next 18 months; not enrolled in another HIV treatment intervention; and currently own or have access to a cell phone, private tablet, or laptop. Potential participants are provided information about the study, consented, and a blood sample for the viral load is obtained (if no recent viral load within 3 months was documented in the medical chart). Participants with an HIV-1 viral load>200 copies/mL are eligible to participate in aim 2. We aim to recruit 240 YBLMSM or YBLTW living with HIV who are not virally suppressed.

##### Study Procedures

After enrollment, participants complete a baseline survey and are randomized (1:1) to 1 of 2 study arms: coach-based MEI or SOC. Randomization to the intervention arms is stratified by site; age group, to yield a balanced randomization between younger (aged 15-19 years) and older (aged 20-24 years) age groups; and enrollment site.

##### SOC

Participants randomly assigned to the SOC arm are provided information about and referrals for HIV care and/or prevention. They return to the research setting every 3 months to complete surveys and conversation with assigned study personnel. The focus is on active listening, with no attempts to direct goal setting; screening, brief intervention, and referral to treatment (SBIRT); or motivational interviewing (MI), as discussed below. Personnel are encouraged to refer to local clinical resources, help with participant care navigation, and case management services at each follow-up visit, as needed.

##### Coach-Based MEI

The coached-based MEI is a tailored and enhanced case management strategy that serves as the primary intervention for participants with unsuppressed HIV viral load. Individuals randomized to the coached-based MEI receive weekly telephonic and app-based support and a structured face-to-face meeting every 3 months, augmented by the ongoing implementation of the study’s mobile app. Telephonic and in-person supportive consultations are goal focused and designed to enhance linkage, ART initiation, adherence, and retention in care for MSM diagnosed with HIV [74-76]. The MEI coach is trained in and utilizes Rogerian listening skills, MI, SBIRT for substance use, and personal health goal setting to best support assigned participants [77-81]. It is based on the case management protocol 078 of the HIV Prevention Trials Network (HPTN) [82], emphasizing routine engagement around health behaviors and goals. To meet the needs of youth, the team modified the approach to adapt it for YBLMSM and YBLTW living with HIV and to incorporate the mobile app and MI components.

##### Training and Selection

MEI coaches were hired based on previous work experience in the MSM community, HIV services (eg, outreach, testing, and case management), interest in aiding research, and ability to communicate effectively with the target population. Representation of the MSM, Black, and Latinx communities was encouraged in the job posting process.

MEI coaches received an initial intensive 16-hour training including (1) introduction to study (2 hours); (2) developmental considerations in cognitive and emotional processing in teens and young adults (1 hour); (3) barriers to PrEP uptake, HIV ART adherence, and HIV risk reduction (1 hour); (4) SBIRT, including screening tools for alcohol and drug use, and effective interviewing about substance use (1 hour); (5) discussion on cultural competence and social determinants of health in case management with YBLMSM and YBLTW (1 hour); (6) mobile app and encouraging engagement with participants (1 hour); (7) study fidelity and data recording expectations (2 hours); (8) didactics and group practice on goal setting and health behavior change (1 hour); and (9) MI components on MI spirit, stages of change, core skills (eg, guiding, open-ended questions, affirmations, reflections, and summarizing), collaborative goal setting, *rolling with resistance*, and repeated role-play (6 hours).

MEI coaches receive ongoing training and support during their continued involvement in the study. Bi-weekly, hour-long videoconference supervision on MI application, goal setting, and troubleshooting are provided remotely by a psychologist with expertise in MI training, substance use, and HIV care. In-person, telephonic, and app-based participant communications, successes, and barriers are discussed as well as topical trainings based on coach needs, MI skills, goal setting, and substance use. The MEI coaches routinely audio-record face-to-face visits with participants assigned conditions, and the supervising psychologist provides individualized written and verbal feedback on each recording on the use of MI and goal-setting techniques, as applied to the individual’s circumstances as well as fidelity to the study intervention.

##### Study Intervention

The intervention is administered by a trained MEI coach and includes substance use screening brief interventions and ongoing monitoring throughout the prevention and treatment care cascade [72]. Coaches begin in the initial face-to-face visit with building rapport, explaining the intervention, and identifying personal and health goals. Health goals focus on medication adherence, health care engagement and satisfaction, substance use, and sexual health. Coaches then map out and help the participant prioritize each goal. After the initial question-answer period and establishment of goals, the MEI coach develops an individually tailored plan that is designed to utilize self-determination theory [83] and the Integrated Theory of Health Behavior Change [84] to facilitate the adoption and maintenance of health behaviors. The approach is centered on identified participant goals and values and care engagement, utilizing a card-sort procedure to help prioritize participant goals for each session [77]. Participants who score positive on the CRAFFT (car, relax, alone, forget, friends, and trouble) and Alcohol, Smoking, and Substance Involvement Screening Test are provided with prevention psycho-education around substance use risks [72,73,85]. Participants who score 2 to 3 on the CRAFFT are encouraged to develop a change plan to reduce substance use and scheduled for additional intervention sessions. These cutoffs were used because a CRAFFT score of 2 or higher is optimal for identifying any substance-related problem and an SUD (according to the Diagnostic and Statistical Manual of Mental Disorders, 5th Edition criteria) [86]. Coaches subsequently attempt to link participants who score above 4 to substance use services. Participants who describe sexual risk (eg, condomless anal sex) are also provided psycho-education around sexual risk and PrEP. Coaches help participants develop strategies to avoid substance use and sexual risks in the future.

At the core of the coached-based MEI approach are supportive services such as navigation, adherence counseling, and tailored support for care engagement and treatment adherence for persons living with HIV. This intervention focuses on 3 steps in the support process: MyCare (steps 1 and 2) and MyHealth (step 3). Participants who are living with HIV start with MyCare (step 1) to support the decision to (re-)initiate HIV care. This includes the use of MI to help orient the client toward seeking and initiating care. MyCare (step 1), which is modeled after life steps, a single-session intervention grounded in cognitive-behavioral principles, is at the core of several successful ART adherence interventions [12,87,88]. MyCare (step 2) adds to this evidence-based approach by recognizing that adherence to medications is a personal choice grounded in a variety of behavioral and psychosocial domains. MyHealth (step 3) is based on the premise that a patient engaged in self-management and supportive strategies will feel empowered to take ownership of their own health care needs.

The face-to-face visits consequently address psycho-education about HIV risk, HIV and ART, planning and problem-solving to access and maintain a steady supply of medication, formulating a daily medication schedule, cues for pill-taking, coping with side effects and/or other participant concerns, developing a plan to mitigate side effects and responses to slips in adherence (if applicable), and engaging around condom use and/or limiting sexual risk behaviors. Counseling is tailored to the participants’ specific needs and case management or navigation needed.

Telephonic and app-based check-ins between the MEI coach and participants are conducted to maintain rapport, open communication, review progress on personally identified goals, help troubleshoot challenges that may arise, and provide reminders for upcoming face-to-face visits. The app is used to facilitate communication around medication adherence, medical visit completion, video chat, and bidirectional communication. The MEI coach will determine the participant’s desire for support, and the frequency and schedule of messages. It is the aspirational goal of the study to have the MEI coach have weekly contact with participants; however, this is often not realistic for all participants. For example, some participants will embrace weekly check-ins, others prefer to engage more intermittently, and the remaining participants may experience situational or motivational barriers that may interfere with engagement. MEI coaches may need to utilize MI strategies to explore readiness to engage in telephonic check-ins and study visits and troubleshoot barriers that may arise (Table 1), and re-engagement needs to be pursued by the MEI coach. MEI coaches seek to check-in with participants weekly (at a minimum), and participants are encouraged to attend face-to-face follow-up coach visits at 3, 6, and 12 months. During these face-to-face visits, coaches will work with the participant to review, motivate, and reinforce personal life events, health care engagement, review substance and risk reduction goals, and problem-solve. Using this MI approach, the participant is thus empowered to take the lead with recommendations and guidance from the MEI coach. Permission is sought to audio-record each initial and follow-up study visit for the purpose of assessing study fidelity and providing routine, ongoing feedback to the MEI coach. The recording is at the discretion and informed consent of the participant and the MEI coach. Ad hoc telephonic and in-person support is also provided by the MEI coach, as requested by the participant. An MEI coach, at any time may help, for example, with referral to behavioral health, job resources, community support resources (eg, a food bank), provide supportive listing, or help arrange any requested sexually transmitted infection testing.

**Table 1:** Motivational interviewing approaches to address level of interest in telephonic engagement.

Participant comments	Stage of change	MEI^a^ coach approach
Agreement: “Sure, I wouldn’t mind texting each week.”	Action	Reinforcement, summarize: “Great, I will make sure I will check-in by text each week, or more if wanted by you.”
Barrier: “That would be good, but my phone sometimes cutoffs if I don’t make my bill.”	Determination	Summarize, empower: “I understand. I will try to text each week, and occasionally you may not have cell service. Any ideas of what we could do to stay in touch if your phone loses service?”
Ambivalence: “I’m not sure, I mean I wouldn’t mind, I guess.”	Contemplation	Paraphrase, elicit, affirm: “I hear you might be willing to consider this, and you might not be sure… (Pause...momentary wait...and follow-up if no response)… I have definitely worked with some people who were wary about the check-ins, is there anything about this you might not be thrilled about?”

^a^MEI: mobile-enhanced intervention.

##### Fidelity

Face-to-face coached-based MEI visits will be recorded. A standardized fidelity checklist will be utilized by the supervising psychologist to quantify discussion of topic domains, rating the domains on a 3-item Likert scale (0=none, 1=partial discussion, and 2=focused discussion) with provided anchors. The domains for coverage during an MEI coach visit are (1) taking medication as prescribed, (2) attending medical appointments, (3) experience at the health provider’s office, (4) encouraging participants’ use of the MEI mobile app, (5) participant’s actual engagement with the mobile app, (6) alcohol or drug use, (7) sexual health, and (8) role of values in participants’ health decision making. A total of 20% of all recorded visits will be reviewed for overall fidelity, seeking a representative sample across MEI coaches, and follow-up visits (eg, baseline and 3-months) across the course of the study. Interrater reliability will be conducted by a coworker of the supervising psychologist trained in the intervention and research methods. MEI coaches will also complete their own fidelity assessment after each MEI coach face-to-face visit, which will help to regularly refresh and reinforce the core elements of each study visit. Data will also be collected from study participants about what topics were covered in their study visits. MEI coaches will also log all study contact with each participant on a secured study record, allowing for monitoring and feedback on the frequency of telephonic encounters.

#### Coach-Based MEI for Youth at Risk for HIV

The third aim is to use an RCT to examine the efficacy of a mobile-enhanced coach-based intervention to achieve uptake of and adherence to PrEP and other preventive behaviors (eg, *protected* sex acts) among youth at elevated risk of HIV infection.

#### Aim 3

##### Study Sample

This sample will be composed of YBLMSM or YBLTW recruited into aim 1, who test negative for HIV but are not currently on PrEP, and report either engaging in condomless anal intercourse or a prior history of a sexually transmitted infection. Additional eligibility includes no plan to relocate in the next 18 months; not enrolled in another PrEP behavioral intervention; and currently own or have access to a cell phone, private tablet, or laptop. Potential participants are provided information about the study and must consent to participate. We aim to recruit 225 YBLMSM or YBLTW at risk for HIV.

##### Study Procedures

After enrollment, participants complete a baseline survey and are randomized (1:1) to 1 of 2 study arms: coached-based MEI or SOC. Randomization to the intervention arms is stratified by site; age group, to yield a balanced randomization between younger (aged 15-19 years) and older (aged 20-24 years) age groups; and the enrollment city. After each participant is identified, they are provided with their assigned treatment assignment.

### SOC

Participants randomly assigned to the SOC arm in the at risk for HIV group receive a similar structure as those with uncontrolled HIV. Initially, they are provided information about HIV prevention strategies and the schedule for follow-up data assessments. During quarterly meetings, a brief check-in conversation and active listening is conducted without the use of SBIRT, MI, or goal-setting, and participants are referred, if needed, to local clinical or community assistive and services at each follow-up visit.

### MEI Coach Intervention

The focus of the Providing Unique Support for Health (PUSH) study is to engage individuals at risk for HIV into prevention and care. The approach is the same as aim 2, modeled after life steps [12,87,88] and based on the case management protocol 078 of the HPTN [82]. It is tailored to the prevention needs of the youth. As such, during the first step, the coach uses MI to orient participants and increase their readiness for preventive engagement. The counseling session addresses psycho-education about HIV risk; planning and problem-solving for mitigating risk, including addressing coexisting substance use; coping with side effects and adherence of PrEP; and/or other participant concerns, developing a plan to address slips in PrEP adherence (if applicable) or condom use. Coaching is tailored to the participants’ specific needs and case management or navigation needed. As with the second aim, randomly assigned participants will be requested to meet with their MEI coach every 3 months and have weekly telephonic check-ins, to review progress on personal goals, risk reduction, and help to troubleshoot factors that may increase HIV risk or personal goals. The MEI coach will utilize MI, SBIRT, active listening, and goal-setting in face-to-face visits, and telephonically will check-in on their lives, their goal progress, and app usage. MI strategies will be utilized to address motivational and situational barriers to study engagement in person or telephonically (Table 1).

Similar to the second aim, this MEI coach intervention focuses on 3 steps in the support process: MyCare (Steps 1 and 2) and MyHealth (step 3). MyCare (Step 2) adds to this evidence-based approach by recognizing that uptake of PrEP or prevention services is a personal choice grounded in a variety of behavioral and psychosocial domains. MyHealth is based on the premise that a patient engaged in self-management and supportive strategies will feel empowered to take ownership of their own health care needs. The MEI coach will determine the participant’s desire for support and the frequency and schedule of messages. For example, some participants embrace engagement intermittently, whereas other participants delay engagement and need to be taken through the engagement steps again. Participants are provided with as much or as little coaching as needed using this flexible approach, but the MEI coach will check-in with participants telephonically weekly (at a minimum), and participants are requested to attend face-to-face follow-up coach visits at 3, 6, and 12 months. During these visits, the coach will review participant’s progress toward personal and health goals, motivate and reinforce treatment engagement, review substance and risk reduction goals, and help problem-solve any obstacles that may have arisen. Using this approach, the participant is thus empowered to take the lead with recommendations and guidance from the MEI coach. Participants receive ongoing screening for sexual risk and substance use, and the coach’s approach is modified based on self-reported needs.

The study infrastructure for supporting and maintaining the MEI coach will be duplicated for the third aim. MEI coaches will receive remote bi-weekly support, instruction, troubleshooting, and feedback from the supervising psychologist, focusing on the delivery of MI, goal-setting, and SBIRT. Audiotaped recordings will be conducted during face-to-face visits, with the informed consent of the participant and discretion of the MEI coach, and will be reviewed by the supervising psychologist for feedback and assistance to the MEI coach and to track study fidelity. Likewise, participants and MEI coaches will complete fidelity assessments postvisit to reinforce study aims, prevent intervention drift, and provide additional data about the intervention delivered.

#### Data Collection for Aims 2 and 3

The PUSH study uses a built-in randomization engine adapted from previous studies [89]. The allocation between intervention and control arms is determined randomly by the engine, where each round comprises 2 intervention and 2 control assignments that are linked to the stratification factors of site and age group. Once a round is completed, a new round begins.

Participants are asked to participate in research visits at 0, 3, 6, 9, 12, and 18 months (exit interview). This visit schedule aims to ensure frequent contact and to minimize attrition. Laboratories and study-related activities are described in Table 2. Surveys provided at baseline (Textbox 1) are re-administered at each follow-up with additional questions focused on ART, PrEP, barriers, and facilitators to adherence.

**Table 2:** Study visits and associated laboratory tests.

Tasks	Visits					
	Baseline	3 months	6 months	9 months^a^	12 months	18 months
Complete a survey	✓^b^	✓	✓	✓	✓	Exit interview
Provide blood for HIV-1 RNA viral load testing (aim 2) or HIV-1 antibody testing (aim 3)	✓	✓	✓	—^c^	✓	—
Provide urine for drug testing	✓	✓	✓	—	✓	—
Update locator information	✓	✓	✓	✓	✓	—
Meet with MEI^d^ coach (intervention only)	✓	✓	✓	✓	✓	✓
Medical chart review (research staff only)	✓	✓	✓	✓	✓	✓
Compensation, US $	50	50	50	10 check-in via phone; 25 survey	50	25
Participate in an in-depth interview (selected participants)	✓	—	✓	—	✓	—
Compensation, US $	25	—	25	—	25	—

^a^9-month visit constitutes either a brief phone check-in (5 min) or in-person survey (15 min).

^b^Data collected at this time point.

^c^Data not collected at this time point.

^d^MEI: mobile-enhanced intervention.

#### Sample Size Considerations

We aim to recruit up to 2000 YBLMSM and YBLTW to be screened for eligibility for aims 2 and 3. We conservatively estimate that 15.00% of the RDS recruited sample will be HIV-infected and not virally suppressed, given an HIV prevalence of 25% to 40% among YBLMSM and YBLTW across the sites and as many as 60% of HIV-infected YBLMSM are undiagnosed [4,9].

We expect a 15.0% loss to follow-up over 1 year in aim 2. Starting with n=240 will yield an effective sample size of 204 individuals in total, or 102 in each group, available for analysis in aim 2. A total estimate of n=200 will be enough to detect an average increase in the viral suppression proportion over the 3 follow-up visits of 16.0% from 24.0% assumed in the control arm to 40.0% in the coached-based MEI arm (odds ratio, OR 2.03), assuming a power of 85.0%. We expect a slightly greater loss to follow-up (20.0% loss to follow-up) in participants in aim 3. Starting with n=225 will yield an effective sample size of 180 individuals in total or 90 in each group. We estimate that a total of 180 participants (90 per group), and assuming a 15.0% average rate on PrEP in the control arm, we can detect an average increase of 14.0% to 29.0% (OR 2.31) with power 85.0%. Lost to follow-up estimates are based on the team’s prior work with this population.

#### Data Analysis

Aim 1 analyses are descriptive and include baseline characteristics related to sociodemographic characteristics, HIV infection, and sexual behavior of the YBLMSM and YBLTW samples across all sites as well as stratified by site. Descriptive tables with unweighted estimates and Pearson chi-squared tests for significance are used to compare age, web-use and venue attendance, and other characteristics of each recruited seed type.

To compare the efficiency of *seeds* from direct versus indirect seed selection in the recruitment of HIV-positive or at-risk youth, we use the RDS statistical package developed by Schonlau and Liebau [90]. This permits an assessment of the overlap of networks across seed types and across recruitment sites. Efficiency is assessed through recruitment diagnostics, including coupon return rate, homophily, the mean number of recruits, and recruitment depth per seed type. This approach also allows us to assess the time from recruitment activation through the achievement of target sample size and response rates. RDS weights will not be utilized in these analyses, as this aim focuses on descriptive sample proportions rather than producing population-based estimates. We will also examine the networks described by younger (aged 15-19 years) and older (aged 20-24 years) participants recruited from different *seeds* and use similar methods to understand the overlap of age groups across different networks.

The primary outcome for aim 2 is durable viral suppression (HIV viral load<200 copies/mL), and the secondary outcome is retention in HIV care (defined as documented 2 or more follow-up medical visits over 12 months, treated as a dichotomous variable). The primary outcome for aim 3 is self-reported PrEP use over 12 months in PrEP users, and the secondary outcome is self-reported condom use in PrEP nonusers. The overall approach is based on an intention-to-treat analysis with treatment assignment (eg, coached-based MEI vs SOC) being the primary independent variable for all models.

The success of the randomization process is measured by comparing the distribution of participant characteristics between the 2 groups at baseline. If the randomization process does not result in comparable groups, adjustment is made for group differences in the multivariable models. For aim 2, logistic regression is used to examine whether participants who receive MEI coach are more likely to be virally suppressed as well as retained and engaged in HIV care than participants in the SOC arm. Generalized estimating equations (GEE) are used to determine whether treatment assignment predicts differences in retention for the coached-based MEI versus the SOC groups using repeated measurements over a 12-month period. GEE is used for the longitudinal data as it adjusts for the intra-personal correlation of repeated measurements on the same individual and provides an estimate of the standard error that is robust to misspecification of this correlation structure.

For aim 3, to evaluate whether YBLMSM and YBLTW receiving coached-based MEI have greater uptake of PrEP over 12-months, a survival model is used to examine whether intervention assignment is associated with time to PrEP use. GEE is used to determine whether treatment assignment predicts differences in PrEP utilization and adherence over time for the coached-based MEI versus SOC groups using the repeated measures over the 12-month period [91]. For both aims, we explore trends in uptake and adherence to treatment or PrEP for the intervention group compared with the control group by including an interaction term between time from randomization and treatment assignment.

Going beyond the intention-to-treat approach, we will develop multivariate models stratified by age group (aged 15-19 years and 20-24 years) and include potential confounders and predictors such as prior exposure to case management and PrEP, sexual risk (low vs high), and other demographic variables. Additional models will be evaluated for possible dose-response relationships considering a measure of the level of engagement with the intervention and the frequency of app use. We will assess whether receiving coached-based MEI results in decreased substance use severity, by examining changes in mean CRAFFT scores (a measure of substance use severity) over time for the intervention group compared with the control group for each aim. CRAFFT scores will be treated as continuous dependent variables, and the models will adjust for intra-personal correlations between repeated measurements. Multivariate linear regression will be used to test for decreased severity at each follow-up visit, while controlling for identified confounders.

Modeling techniques have been selected based on the capacity to adequately handle the missingness of data. GEE models assume that the missingness is because of a missing at the random mechanism, which is a restrictive assumption. To address dropout, we use inverse probability weighting to relax this assumption of missing at random [92,93]. Other types of missing data will be handled using multiple imputations.

#### In-Depth Interviews

In-depth interviews are completed as part of routine follow-up visits (Table 2). Target enrollment for interviews is 6 participants (3 SOC and 3 MEI) per aim per site (total of 36). This total will be sufficient to reach informational redundancy across the 3 cities [53]. Interviews focus primarily on changes in self-reported sexual orientation and gender identity, mentorship and community experiences, health care navigation, barriers or facilitators to care or research, and substance use specific to YBLMSM and YBLTW. Initial analysis of the interview data follows the same procedure outlined in the formative data section.

Further analysis will allow for responses and interview themes to be compared across sites, time, participant’s age, and other sociodemographic characteristics. The findings are presented to the team on an ongoing basis to provide insight into barriers and facilitators of engagement and intervention experiences over time. Participants are paid US $25 for each interview.

#### Consent and Human Subjects Considerations

Participants in this study go through a verbal consent process (paper or electronic) to enter the formative research, RDS survey, and/or enroll in the RCTs. Electronic consent displays on the screen before the web-based survey. Participants are prompted to provide their signature on the screen as an acknowledgment of consent. This study has obtained a waiver of parental permission for unaccompanied participants and adolescents seeking confidential services who are aged 15 to 17 years under 45 CFR Part 46.408(c), which is in line with local laws in (Maryland HG Section 20-102), Pennsylvania law (IRB SOP 505; Minors’ Consent Act, 35, PS 10101), and Washington DC law (code 600.7). For youth aged 15 to 17 years recruited through RDS that are not seeking confidential services, we will use *advocates* (trained social workers) who are independent counselors to work with each participant aged 15 to 17 years before and during the informed consent to ensure adequate comprehension of risks and benefits of participating in the research in Baltimore.

To provide adolescents with additional protections, a certificate of confidentiality has been obtained from the National Institutes of Drug Abuse (NIDA). All staff are trained on confidentiality, HIPAA (Health Insurance Portability and Accountability Act) privacy protections, cultural competency, adolescent counseling techniques, pre- and posttest counseling, mandated reporting, statutory rape laws (specific to the local context), and managing situations of child or sexual abuse. Adolescents who describe physical or sexual abuse or express concerns about mental health are linked to child protective or social work services to ensure their safety. This study was approved by the CHOP, the Children’s National Hospital, and the Johns Hopkins School of Public Health institutional review boards.

## Results

A total of 585 participants have been screened to date, with 450 (76.9%) enrolled. Recruitment monitoring showed that seed propagation was slow and reached low depths, frequently ranging from 1 to 3 waves of recruitment before stopping.

Table 3 summarizes the key demographic characteristics and reported substance use of baseline participants (N=402) and those who enrolled in aim 2 (n=36) and aim 3 (n=123). In summary, the median age was 21.6 years at enrollment, most participants identify as African American/Black (76.6%), 41 (10.2%) identify as transgender or female, 145 (41.5%) completed high school, and 145 (36.1%) had at least some college or technical education. One-quarter (97/402, 24.1%) reported having been without a place to stay in the last year, and most participants reported a history of substance use: alcohol (77.6%); cannabis (76.6%); and about a fifth reported use of other drugs including opioids, amphetamine, and cocaine (21.9%). Almost a quarter (n=100) reported engaging in transactional sex for a place to stay, money, or food. Moreover, 10% of the participants described having been forced to do something sexually (coerced sex, n=40) and 10.8% (n=43) described being forced not to use a condom during sex. Qualitative research is also ongoing. A total of 19 baseline interviews have been conducted within the 3 sites, with 9 interviews from aim 2 participants and 10 interviews from aim 3 participants.

**Table 3:** Demographic characteristics and substance use at baseline for participants.

Characteristics		AMSAB^a^ participants (N=402)	Participants in aim 2 (n=36)	Participants in aim 3 (n=123)
Age (years), median (IQR)		21.6 (19.3-23.5)	22.9 (19.8-23.9)	20.7 (18.8-22.8)
**Age category (years),** **n (%)**				
	15-17	42 (10.4)	4 (11.1)	17 (13.8)
	18-24s	360 (89.6)	32 (88.9)	106 (86.2)
**Race,** **n (%)**				
	Black/African American	308 (76.6)	30 (83.3)	91 (74.0)
	Black/mixed	28 (7.0)	1 (2.8)	6 (4.9)
	Latino/Black	26 (6.5)	3 (8.3)	12 (9.8)
	Hispanic	30 (7.5)	0 (0.0)	9 (7.3)
	Unknown	10 (2.5)	2 (5.6)	5 (4.1)
**Gender,** **n (%)**				
	Male	347 (86.3)	30 (83.3)	108 (87.8)
	Transgender	28 (7.0)	3 (8.3)	8 (6.5)
	Female	13 (3.2)	2 (5.6)	1 (0.8)
	Gender nonbinary or nonconforming	13 (3.3)	1 (2.8)	6 (4.8)
**Sexual identity,** **n (%)**				
	Gay	255 (63.4)	25 (69.4)	86 (69.9)
	Bisexual	88 (21.9)	5 (13.9)	24 (19.5)
	Heterosexual	25 (6.2)	1 (2.8)	5 (4.1)
	Queer	10 (2.5)	0 (0.0)	4 (3.2)
	Pansexual	9 (2.2)	2 (5.6)	2 (1.6)
	Other or questioning or missing	33 (3.2)	3 (8.3)	2 (1.6)
**Highest level of education,** **n (%)**				
	<High school	89 (22.1)	12 (33.3)	29 (23.6)
	High school graduate	167 (41.5)	13 (36.1)	50 (40.6)
	>High school	145 (36.1)	10 (27.8)	44 (35.8)
**Are you employed?,** **n (%)**				
	Yes	227 (56.5)	17 (47.2)	71 (57.7)
	No	174 (43.3)	18 (50.0)	52 (42.2)
**Been without a place to stay in the last 12 months,** **n (%)**				
	No	301 (74.9)	26 (72.2)	103 (83.7)
	Yes	97 (24.1)	9 (25.0)	20 (16.3)
**Substance use ever,** **n (%)**				
	Tobacco	154 (38.3)	26 (72.2)	35 (28.5)
	Alcohol	312 (77.6)	29 (80.6)	97 (78.9)
	Cannabis	308 (76.6)	30 (83.3)	100 (81.3)
	Any other drugs (amphetamine, cocaine, opioids, sedatives, or inhalants)	88 (21.9)	15 (41.7)	27 (22.0)

^a^AMSAB: assigned male sex at birth.

## Discussion

### Principal Findings

The lack of data focused on identifying, recruiting, and engaging YBLMSM and YBLTW who are living with or at risk of acquiring HIV and have high rates of substance use contribute to health disparities and a public health crisis in this population.

Previous studies have described the use of RDS as an effective strategy to reach hard-to-reach populations. Some have also demonstrated challenges (eg, slow propagation) in reaching YBLMSM and YBLTW, though these studies have failed to examine different seed selection methods used in RDS [94]. The PUSH study allows us to examine assumptions about RDS seed selection methods and barriers to participating in research, which may help to inform future recruitment and sampling methods for interventions with sexual and gender minority youth.

This intervention study collects critical data on whether modification of an existing model for persons living with HIV can be used for YBLMSM and YBLTW living with HIV and those at risk for HIV. Evaluation of the PUSH study will address multiple gaps, including what components (eg, mobile app, telephone, and coach support) are needed to improve youth-based interventions to be more accessible and able to simultaneously address HIV treatment or prevention and substance use care. Although peer support is thought to be an additional strategy to improve access to care in this population [95-97], the gap in existing knowledge about the impact of coaches and/or peer-based strategies leaves prevention and treatment programs uncertain about their efficacy and effectiveness.

### Limitations

There are limitations noted in this protocol. The focus population is only YBLMSM and YBLTW, limiting the generalizability of the findings. The focus groups were small and completed in 1 city, with feedback from 1 youth advisory board. We further clarified areas identified from focus groups in in-depth interviews across the cities, but this intervention may not be reflective of all the needs of YBLMSM and YBLTW. The protocol sample size estimates are based on high retention estimates.

Despite these limitations, the results of this study have the potential to significantly impact the medical and substance use services provided to YBLMSM and YBLTW in the United States by providing rigorous scientific evidence outlining approaches and strategies to improve uptake and engagement in HIV and prevention cascade. Despite accounting for disproportionate rates of HIV, most interventions in youth do not focus only on youth of color. This study focuses solely on youth of color in 3 urban cities. A major strength of the PUSH study is that it further provides information about the use of a multi-city approach to engage youth of color, along with exploring similarities and differences across locations. This work will provide data on the prevention, treatment, and sexual health trajectories of YBLMSM and YBLTW, including how to promote facilitators, address barriers to engaging in care, and end the HIV epidemic in this population.

## Abbreviations

ART: antiretroviral therapy
CHOP: Children’s Hospital of Philadelphia
CRAFFT: car, relax, alone, forget, friends, and trouble
e-coupons: electronic coupons
eRDS: electronic respondent-driven sampling
FGDs: focus group discussions
GEE: generalized estimating equations
HPTN: HIV Prevention Trials Network
KI: key informant
KIIs: key informant interviews
LGBTQ: lesbian, gay, bisexual, transgender, queer
MEI: mobile-enhanced intervention
MI: motivational interviewing
MSM: men who have sex with men
NIDA: National Institutes of Drug Abuse
OR: odds ratio
PrEP: pre-exposure prophylaxis
PUSH: Providing Unique Support for Health
RCT: randomized controlled trial
RDS: respondent-driven sampling
SBIRT: screening, brief intervention, and referral to treatment
SOC: standard of care
SUDs: substance use disorders
TW: transgender women
YBLMSM: young Black and Latinx men who have sex with men
YBLTW: young Black and Latinx transgender women
